# Mesenchymal Stem Cell (MSCs) Therapy for Ischemic Heart Disease: A Promising Frontier

**DOI:** 10.5334/gh.1098

**Published:** 2022-03-03

**Authors:** Merlin Sobia Poomani, Iyyadurai Mariappan, Ramachandran Perumal, Rathika Regurajan, Krishnaveni Muthan, Venkatesh Subramanian

**Affiliations:** 1 Department of Biotechnology, Manonmaniam Sundaranar University, Tirunelveli 627012, Tamil Nadu, India; 2 Trichy Medical Center and Hospital, Tiruchirapalli 620018, Tamil Nadu, India; 3 Center for Marine Science and Technology, Manonmaniam Sundaranar University, Tirunelveli 627012 Tamil Nadu, India

**Keywords:** ischemic heart disease, mesenchymal stem cells, cardiac regeneration

## Abstract

Although tremendous progress has been made in conventional treatment for ischemic heart disease, it still remains a major cause of death and disability. Cell-based therapeutics holds an exciting frontier of research for complete cardiac recuperation. The capacity of diverse stem and progenitor cells to stimulate cardiac renewal has been analysed, with promising results in both pre-clinical and clinical trials. Mesenchymal stem cells have been ascertained to have regenerative ability via a variety of mechanisms, including differentiation from the mesoderm lineage, immunomodulatory properties, and paracrine effects. Also, their availability, maintenance, and ability to replenish endogenous stem cell niches have rendered them suitable for front-line research. This review schemes to outline the use of mesenchymal stem cell therapeutics for ischemic heart disease, their characteristics, the potent mechanisms of mesenchymal stem cell-based heart regeneration, and highlight preclinical data. Additionally, we discuss the results of the clinical trials to date as well as ongoing clinical trials on ischemic heart disease.

## Introduction

Ischemic heart disease (IHD), a life-threatening cardiac condition characterised by lessened blood and oxygen supply to the heart, which is the principal component of cardiovascular diseases (CVD), may lead to heart failure and myocardial infarction (MI) [1,2]. It is the major cause of death in urbanised nations and contributes significantly to the disease burden in developing nations [3]. Heart failure is prevalent, particularly in industrialised countries; it affects between 3% and 5% of the population and has developed into an evolving disease with high diagnostic costs [4]. For cardiac viability and optimal function, equity between myocardial oxygen quantity and demand is required [5]. Myocardial ischemia occurs when mitochondria fail to supply the energy demand due to deficient oxygen supply when the myocardium shifts to anaerobic glycolysis to generate energy [6]. As a result of anaerobic conditions, myocardial ability to produce adequate oxygen to sustain cardiomyocellular processes is limited. In turn, oxygen deprivation results in decreased nutrient availability and insufficient removal of metabolic end products. This process changes the series of events in cardiomyocytes’ biology and cardiac structure that occur in endothelial cells, fibroblasts, vascular smooth muscle cells, and leukocytes [7]. It is the major reason for ischemic conditions and also for ischemic heart failure.

Current therapies include inhibition of the angiotensin-altering enzyme, beta and aldosterone barriers, and biventricular striding [8,9]. However, mortality and morbidity [10,11] from HF cause these strategies to succeed only temporarily, necessitating novel strategies to inhibit and reverse cardiac dysfunction. Cell-based therapy became popular in the 1990s and is promoted as a promising field in regenerative medicine for repairing cardiac damage after MI [12]. Mesenchymal stem cells (MSCs) have occurred as a breakthrough treatment to “regrow” lost cardiomyocytes and repair endogenous tissue by paracrine signalling and exhibit novel immunomodulatory properties [13]. Subsequently, the success of MSCs in cardiac disease has been the intensity of research [14]. In this review, we will focus on the use of mesenchymal stem cells and their paracrine effects in MI to reverse dysfunction and review clinical trials using this novel regenerative approach. The ease of isolation, expansion, and *in vitro* multilineage potential positioned MSCs as promising therapeutics in regenerative medicine.

## 1. Discovery of mesenchymal stem cells

A half century ago, Friedenstein and co-workers discovered that bone marrow is not only the source of hematopoietic stem cells but also the reservoir of mesenchymal stem cells in adult organisms. They isolated mono-layered fibroblast-like bone-forming cells or colony-forming cells-fibroblast (CFU-F) from guinea pigs [15,16,17], and Owen expanded on this work in rats [18]. However, the isolation and culturing of human bone marrow MSCs were reported in 1992 [19]. Friedenstein showed that these cells are multi-potential, self-maintenance precursor cells and can differentiate into osteocytes as well as chondrocytes and adipocytes *in vitro* [15,16]. The phrase “mesenchymal stem cells” was suggested by Arnold Caplan in 1991 due to their differentiation capability into more than one form of cell that form combinative tissue in many organs [20,21]. This name was commonly used even though it had qualms about its stemness [22].

## 2. Criteria for MSCs by ISCT

**Figure 1:**
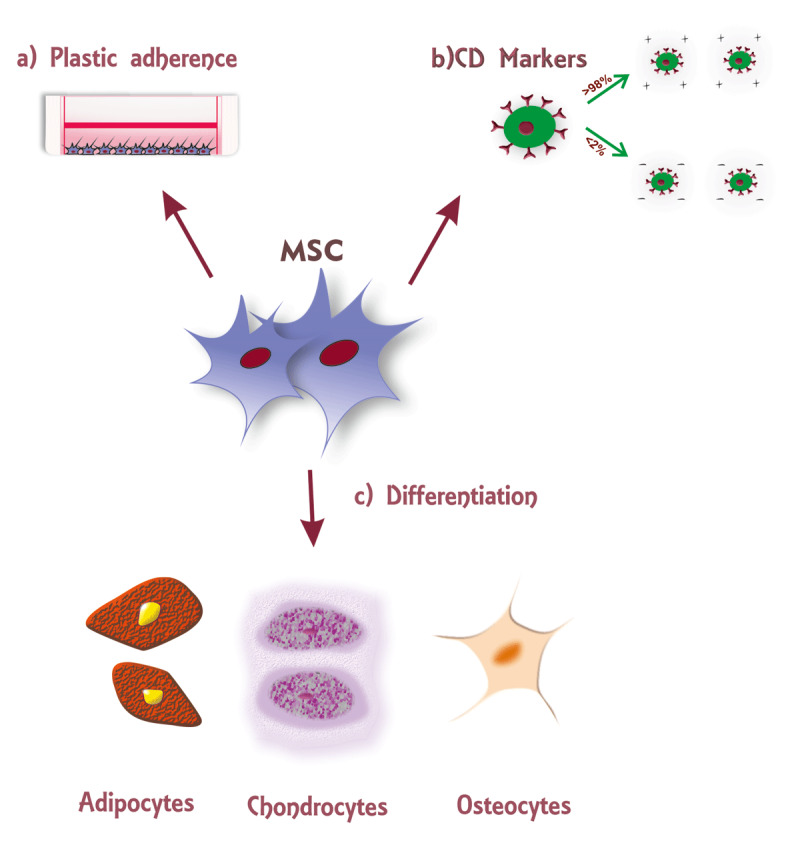
According to the International Society for Cellular therapy (ISCT) statement, Mesenchymal Stromal Cells defined as **a)** adherent to the plastic surface **b)** express Cluster Differentiation antigen, >98% positive for CD105, CD73, and CD105, and <2% negative for CD45, CD34, CD14 or CD11b, CD79alpha or CD19, HLA-DR surface molecules c) should differentiate into adipocytes, chondrocytes, and osteocytes.

In 2006, the International Society for Cellular Therapy (ISCT) issued the criteria to define the population of MSCs to halt the growing controversies regarding the definition of MSCs, nomenclature, degree of stemness, and characteristics of cells. To define human MSCs, the proposed minimal criteria are that 1) they should be plastic adherent while maintaining incultured conditions; 2) they must express positive markers such as CD105, CD73, and CD90 while expressing negative markers such as CD45, CD34, CD14, or CD11b, as well as CD19 and class II histocompatibility complex antigens (HLA-DR); 3) they should differentiate into adipocytes, osteocytes, and chondrocytes [23]. The pictorial representation of the criteria for mesenchymal stem cells is represented in ***Figure 1***.

By fulfilling the criteria, mesenchymal stem cells can be reaped from numerous tissues at all progressive stages, such as fetal, young, adult, and old. Various reports have been published on the possible sources for isolating the cells. The most common and large utilised adult tissues are bone marrow [19,24] and adipose tissue [25,26,27]. Then the young adult tissues such as umbilical cord tissue [28,29] and placenta [30,31] from which juvenile MSCs can be isolated noninvasively. The other sources include dental pulp, yellow ligament, peripheral blood, endometrium, fetal tissues such as amniotic membrane, chorionic villi, and Wharton jelly, which are used to obtain MSCs [32,33,34,35,36,37,38,39,40,41].

## 3. Mesenchymal stem cells in cardiac repair

The clinical use of MSC started between 1995 and 2000, especially for the treatment of cancer and bone/cartilage disease [42,43,44]. Later, the outcome of several clinical works adjudged the therapeutic potential as well as safety of using MSC for treatment of various ailments. Consequently, clinical trials have also established the virtue of MSC in the treatment of acute myocardial ischemia (AMI) [45]. However, to achieve the purpose of cardiac regeneration, the desired progression must include three objectives: 1) the mass production of additional myocardial mass, 2) the development of a new vascular system to withstand it, and 3) the replacement of a weakened ventricle to its suitable geometry [46,47]. Amongst all, Pittenger et al. (1999) directed attention to mesenchymal cells procured from bone marrow and emphasised that these cells proliferated extensively *in vitro* conditions and could be an attractive candidate for transplantation [24]. In 2001, an evolving study revealed that BM-derived cells from the murine model infused intramyocardially improved cardiovascular function after myocardial infarction (MI) (48%). After that, in 2004, Chen et al. (2004) demonstrated the intracoronary infusion method in autologous BM-derived MSCs improved left ventricular (LV) function [49]. The key property of MSCs is that they lack major histocompatibility complex II (MHC-II) markers, that is, they elude rejection by the immune system (both innate and adoptive) [50]. MSCs also have the characteristics of anti-fibrotic and anti-inflammatory properties [51]. These reports suggest that allogeneic use of these cells could be valuable. Accordingly, in randomised clinical trials, the transendocardial injection of both allogeneic and autologous derived MSCs in ischemic patients resulted in a favourable response in immunological reactions, the eminence of life, and ventricular remodelling [52]. A few scientists studied patients with heart failure caused by severe ischemic conditions in randomised, placebo-controlled trials. After a six-month study, MSC-treated patients revealed a substantial decline in LV end systolic volume and an increase in LV ejection infarction [53]. The success of these efficient studies made cardiac regeneration by cell-based therapy via mesenchymal stem cells more appealing in the research field.

## 4. Potent mechanisms proposed for cardiac repair

**Figure 2:**
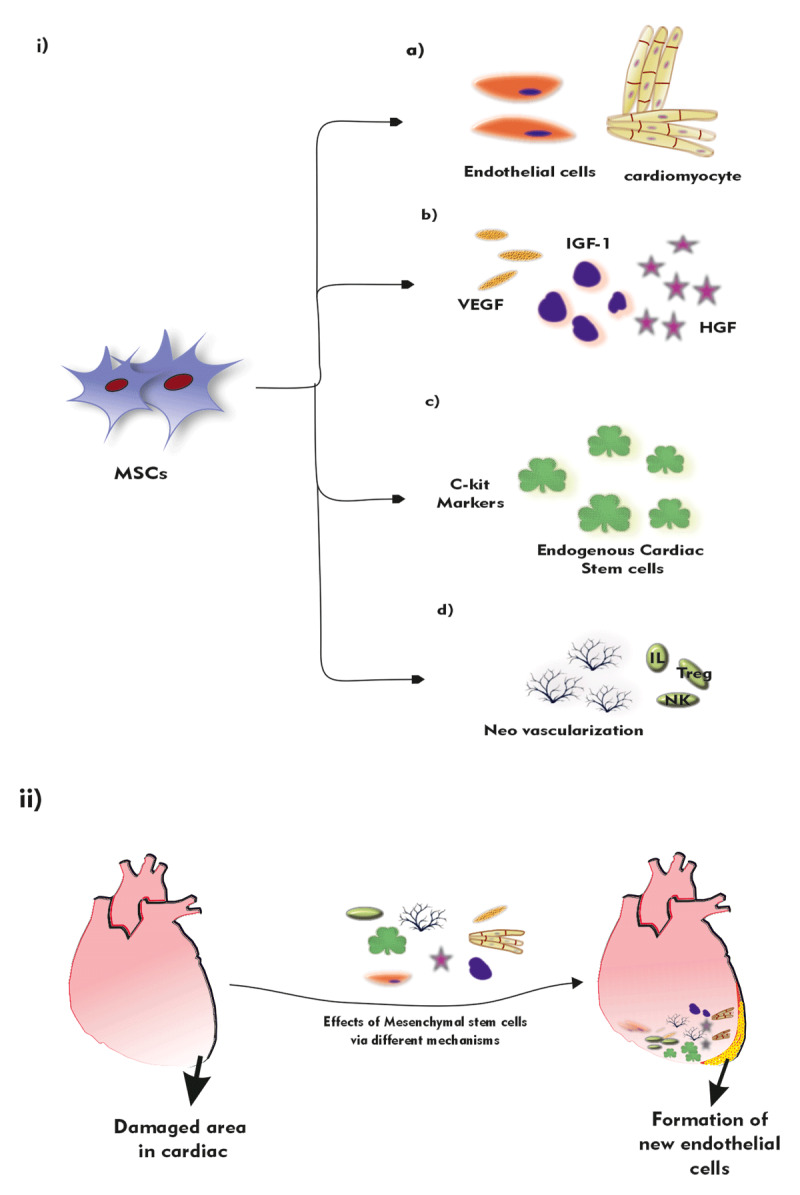
Mechanism of action of Mesenchymal stem cells: I) MSC cells can **a)** transdifferentiation into endothelial cells and cardiomyocytes **b)** promote paracrine signals such as Vascular endothelial growth factor (VEGF), Hepatic growth factor (HGF), and Insulin-like growth factor (IGF) **c)** proliferate endogenous cardiac stem cells with C-Kit markers **d)** stimulate the growth of neovascularization and immunomodulation. II) Overview of formation of new endothelial cells, reduction in infarct size of damaged cells via various mesenchyme effects.

The precise mechanism of MSC and its actions are still unknown. It is proposed to be numerous effects from these stem cells before opting for a single mechanism of action. Several kinds of research have contributed to the salutary effects of MSC therapy by various factors, both *in vivo* and *in vitro*, after cardiomyocytes. Feasible mechanisms consist of 1) new engraftment and differentiation into new cardiomyocytes or other cells [54,55,56,57]; 2) paracrine signaling/mediators from MSC show adverse effects on cardiac repair [58,59]; 3) MSC stimulate endogenous cardiac stem cells (CSC) to proliferate and repair injured tissues [60,61]; as well as [4] stimulation of neovascularization and immunomodulation [62]. Further research may lead to the precise mechanism of MSC with improved techniques to repair damaged hearts with more effective functions. The different approach mechanisms are illustrated in ***Figure 2***.

These four combined actions are more powerful to inhibit undesirable remodeling of organs damaged by ischemic injury. The predominant disease caused by an ischemic wound to the heart leads to the deprivation of contractile cardiomyocytes and replacing normal tissue with fibrotic scar tissue. To prevent this, the demonstration of MSCs injected into the frontier region of infarcts and applicable cardiac tissue in pre-clinical and clinical trial settings. It resulted in an anti-fibrotic effect, reducing scar size, promoting endogenous tissue regeneration, reduced inflammation, stimulate cellular growth, proliferation, and improving perfused and viable blood vessels to reverse the cardiac injury. The enhanced endogenous mechanism shows improved contractile cardiac muscle than the direct differentiation and engraftment of MSCs [53,63,64,65,66,67].

**Figure 3:**
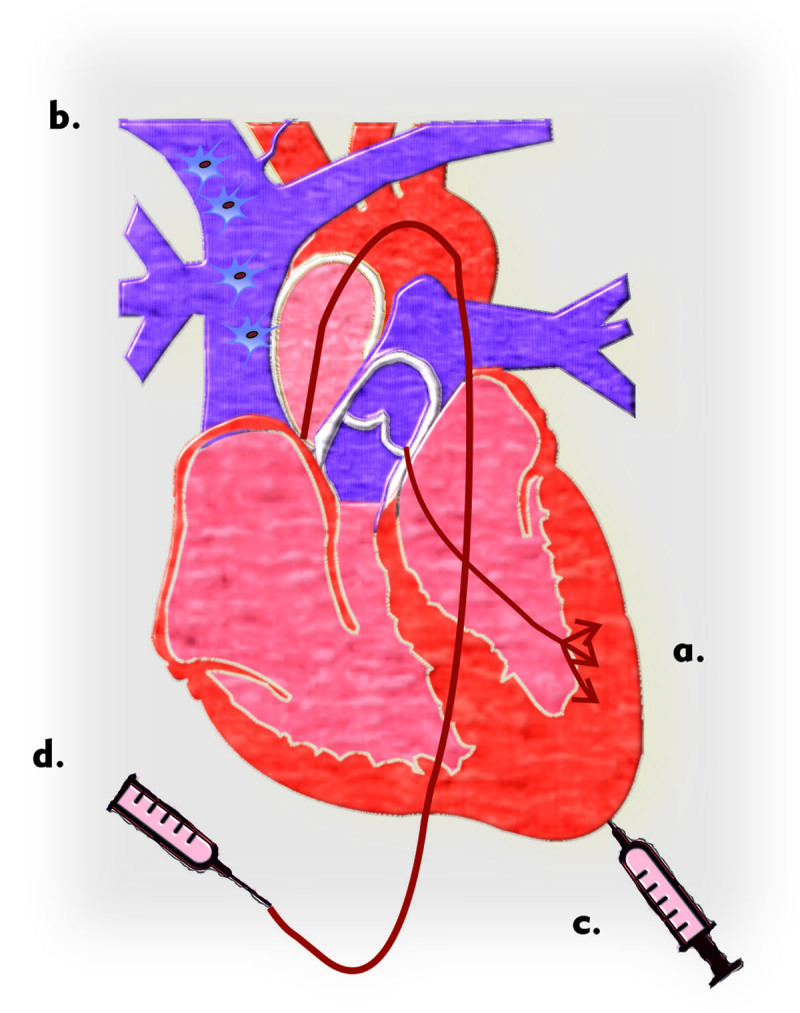
A diagram of various cell therapy approaches tested with MSCs is shown: **a)** transendocardial stem cell injection, **b)** peripheral intravenous infusion, **c)** intramyocardial delivery by injection catheter, and **d)** delivery via an intracoronary artery.

The efficacy of the route of MSC administration needs to be assessed. The effective routes tested with MSCs are depicted in ***Figure 3***. Several methods of MSC delivery *in vivo* have been proposed to improve cardiac recovery, including 1) transendocardial stem cell injection (TESI) [68,69], 2) intravenous infusion at the periphery [70,71,72], 3) injection catheter-based direct intramyocardial (DI) delivery [73,74,75,76], and 4) intracoronary infusion delivery [77,78,79]. The route of administration appears to have a remarkable influence on the effectiveness of MSC therapy in both acute and chronic myocardial infarction [80]. Transendocardial injection shows superiority with condensed infarct size (n = 49, 9.4% level reduction; 95 percent confidence interval, from 15.9 to 30.0) and similarly upgraded left ventricular ejection fraction (LVE) in a systematic review study that examined the impact of MSC therapy in both AMI and chronic dilated cardiomyopathy with various routes of delivery in a swine model and clinical trials, 9.1% level increase and 95 percent confidence interval, 3.7 to 14.5) TESI also improved LVEF in humans (n = 46, 7.0% level increase; 95 percent confidence level, from 2.7 to 11.3), whereas DI, arterial, intracoronary infusion, had no effect in the demonstration. In chronic ischemic cardiomyopathy, the transendocardial with the application of 20–100 × 10^6^ MSCs was given the best results compared to others at the conclusion of cardiovascular clinical trials [81].

## 5. Paracrine effect, extracellular vesicles and exosomes

Even though MSC can act through different mechanisms, the paracrine activity of MSC has been the focus of therapeutic effects in recent studies. Multiple growth factors and cytokines are secreted together as a “secretome”. These factors are soluble in water or encapsulated in extracellular vesicles (EV) or exosomes by culturing the cells in a “conditioned medium” [82,83,84,85]. Interleukin-1 (IL-1) and -6 (IL-6) for angiogenesis promotion and VEGF induction, hepatocyte growth factor (HGF) for cardiac progenitor cell mobilization, transforming growth factor (TGF-) 1 acts as a fibrosis regulator, and placental growth factor (PLGF) and vascular endothelial growth factor (VEGF) work to prevent cardiomyocyte and endothelial cell death [86,87,88,89,90,91]. Angiogenin secretion is thought to function as LV remodelling attenuation via vasculogenesis [92,93,94]. A few mRNAs secrete matrix mediating factors such as matrix metalloproteinases (MMPs) 2, 9, and 14 to surge the antifibrotic action [95,96] and are capable of crossing through type I collagen membranes [93,97]. A novel up-regulated secreted protein, Akt-MSC, induced hypoxic conditions to promote cardiomyocyte survival with reduced infarct size and reduced apoptosis [58,98,99].

In the myocardium, the secretion of paracrine factors has a pleiotropic effect, such as improvement in local angiogenesis, reduced cardiomyocyte death, low fibroblast activation, cardiac stem cell stimulation, and also a reduction in myocardial fibrosis, corresponding with the cell-mediated immune response [100]. Various preclinical research models have shown the secretion of soluble factors by the MSCs in different models. Nakanishi and coworkers reported that the up-regulated expression of cardiac progenitor genes, namely myosin heavy chain and atrial natriuretic peptide (ANP), in a conditioned medium derived from MSCs. This paracrine activation enhanced the migration and differentiation of cardiac progenitor cells (CPC) [101]. According to Cho et al. [102] MSCs after overexpressed glycogen synthase kinase (GSK)-3 were induced into the coronary ischemic heart, resulting in reduced mortality, reduction in infarct size, high yield of cardiomyocyte differentiation rate, and LV modeling. The overexpression of the survival gene Akt1 (Akt-MSC) released *in situ* by MSC restores cardiac function and prevents ventricular remodelling in less than 72 hours. Not only that, but it also acts as a protective paracrine marker for ischemic myocardium [103]. Numerous cytokines were produced from bone marrow-derived stem cells in the *in vivo* model of acute MI, as well as vascular endothelial growth factor (VEGF), interleukin (IL)-1, platelet-derived growth factor (PDGF), and insulin-like growth factor (IGF)-1. These cytokine factors functionally improved the infarcted heart from ischemic injury by the preservation of contractile proportions of the myocardium, inhibited cell death of myocardiocytes, and induced angiogenesis of the infracted heart [104,105,106].

Extracellular vesicles and exosomes are membrane-limited cellular components used for regenerative therapy. Current research focuses on the supernatant of cells that have similar components to MSC. EVs range in size from 100 nm to 1 m and are formed by the detachment of cytoplasmic protrusions [107]. Contrarily, exosomes have a range of sizes between 30 and 100 nm. It leads to the fusion of multivesicular endosomes and plasma membranes released by MSCs, as well as exocytosis [107]. The secretome of EV from MSC expresses CD13, CD29, CD44, CD73, and CD105, depleting the characteristics of MSCs’ origins [108,109,110]. Both carry nucleic acids, coding mRNA and non-coding RNA, which are functionally equivalent. The EVs’ coding mRNA are transcription factors that regulate transcription, cell multiplication, and immune regulation [111]. Amongst the non-coding RNAs that repress released EV-MSC, they have a selective pattern for miRNAs [108,112], which can regulate gene expression post-transcriptionally by targeting cells and downregulating the targeted proteins by highly expressed miRNAs [113].

The latest research suggests the use of EVs and exosomes as the therapeutic paradigm in ischemic conditions. In the enriched exosome of miR-22 secreted from MSCs, the anti-apoptotic effects were pointed out through methyl CpG binding protein-2 (Mecp2) in ischemic preconditioning and *in vivo*, it significantly reduced cardiac fibrosis [114]. Another study on the highly purified exosome from an MSC-conditioned medium with a hydrodynamic radius of 55–65 nm was shown to mediate cardioprotection during myocardial ischemia in a murine model [115]. Successive works from the same group demonstrated that the exosome proteome tends to identify protein candidates that are attributed to the therapeutic effect, demonstrating that the 20S proteosome, as a candidate exosome protein, is responsible for the reduction in the size of the infarct in a mouse model of myocardial ischemia [116]. Likewise, MSC changed over with GATA-4 formed exosomes containing a high concentration of miR-221. This mRNA inhibited apoptosis in ischemic cardiomyocytes by downregulating the expression of p53-stimulated modulator of apoptosis (PUMA), a Bcl-2 protein family subclass [117]. The combinatorial delivery of exosomes (Exo) and MSCs into acute MI via intramyocardial injection enhanced the cardiac function, reduced the inflammatory response, and improved the microenvironment via Exo injection [118].

## 6. Immunomodulation of MSCs after ischemic injury

Mesenchymal stem cells turned up as dominant modulators of both the innate and adaptive immune systems. Numerous MSC-mediated immunomodulatory effects are mediated by host cells. The elimination of cellular debris, activation of resident precursor cells, the compensatory growth of residual cardiac tissue, quantitative and qualitative changes in the vascular network, the development of fibrotic scar, and inflammatory reactions were found to guide the regeneration process after cardiac damage [119]. MSCs have been shown in numerous clinical trials to be immune suppressive for at least 12 months following transplantation [120]. Nonetheless, anti-inflammatory M1 macrophages, soluble mediators such as TGF-1, prostaglandin E2, hepatocyte growth factor (HGF), indoleamine 2, 3-dioxygenase, soluble HLA G5, heme oxygenase-1, and anti-inflammatory interleukin 10 (IL10) are well-known [121]. MSC reduces the stimulating receptors of natural killer (NK) cells in the innate system, and in the adaptive system, they inhibit B cell and dendritic maturation, suppress T helper (Th) cell and cytotoxic T cell (Tc) proliferation, and prevent T-cell production of pro-inflammatory cytokines [122]. In mouse hearts, after the MI’s inception, human MSCs functioned to reduce inflammatory response by secreting TNF-stimulated gene-6 (TSG-6) induced protein intravenously. This study discovered that inducing this molecule was also related to a fall in infarct size, a decrease in the proteolytic wound to the heart, a decrease in fibrosis, and an improvement in cardiac dysfunction [123]. Regulatory T cells (Treg) contributed to statin-induced cardioprotection against ischemia-reperfusion injury through specific depletion, reduced infarct size, and inhibited inflammatory responses [124]. Modification of MSCs with these growth factors and cytokines showed the beneficiaries by improving vascularization and cardiac remodeling, reducing inflammation and enhancing angiogenesis [125].

## 7. MSCs in animal models of MI

Even though the successive characteristics of mesenchymal precursors/stem cells are already illustrated, *in vivo* experiments in animal models of the particular ailment are mandatory to implement those studies into humans. Preclinical studies helped to explore the regenerative capacity of MSCs in small and large animal replicas of acute and chronic ischemic heart ailments [126].

In experimentation, small animals like mice, rats, and rabbits are often castoff as models for cardiovascular diseases due to their small size, simple to handle, low maintenance, and inexpensive cost [127]. The first experiment by Orlic et al. was shown in female mice with an acute MI. Intramyocardial administration of green fluorescent protein (GFP) named lineage c-kit BM-MSC improved cardiac regeneration. They made the assumption that BM-derived stem cells would engraft myocardiocytes, endothelial cells, smooth muscle cells, and vascular structure in the infarct area, repairing myocardium *in vivo* [48] Adipose-derived stem cells (ADSC) implantation in rats after coronary infarction increased the secretion of VEGF, that is, enhancement of angiogenesis and neovascularization under hypoxia conditions after a week of infusion [109]. The transplantation of BM-MSC into the dilated cardiomyopathy (DCM) rabbit model differentiated into cardiomyocytes-like cells and myocardial cells with the induction of 5-azacytidine. Not only did they observe the upregulation of VEGF expression, but they also up-regulated the receptors such as Flt-1 and Flk-1 [128]. The DCM rat model was treated with human umbilical cord (hUC)-MSCs, which repressed the expression of TNF-, extra-cellular signal-regulated kinase 1/2 (ERK1/2), and TGF-1 signalling pathways, resulting in reduced fibrosis and cardiac dysfunction [129]. In preclinical trials, common findings include reduced infarct size, improved LVEF, and vasculogenesis [130]. In addition, improved VEGF expression and blood flow in the infarct zone made the situation more beneficial [126,131].

However, there are some drawbacks to using small *in vivo* animal models, such as minute heart size and anatomical variations in the coronary blood vessels, which result in a high failure rate in clinical transplantation [132]. Animals such as pigs, porcine, dogs, and sheep are utilised in scientific experiments since they are more human-like. Infusing BM-derived MSCs intravenously into pigs with reperfusion/acute ischemia injury enhances left ventricular ejection fraction (LVEF), can enlarge ventricular arrhythmia in myocardial infarction hearts, and limits adverse wall thickening [133]. In another study, the intracoronary infusion of MSC cells into the porcine heart after five days of infarction showed the improvement of increased LVEF and scar reduction in infarct size [134]. Endomyocardial delivery of allogeneic mesenchymal precursor cells via catheter into sheep after four weeks post MI using three-dimensional echocardiography. It showed the inflation in the LV act (ejection fraction and wall stiffening) and neovascularization [135]. The combination of autologous MSC with CSC cell therapy improved myocardial contractile performance by reducing scar size by about 44.1 ± 6.8%, improving EF by about 6.9 ± 2.8 EF units, and also diastolic strain, which exhibits increased cardiomyocyte mitotic activity in a swine model three months after ischemic/reperfusion injury [136]. In a porcine model of severe MI, the intracoronary jab of allogeneic adipose tissue procured-MSC improved myocardial perfusion, reduced infarct size, and enhanced the reparative process in cardiac function after two months [137,138]. Despite this, none of these models can reliably reproduce the complexity and pathology that characterise human myocardial infarction. When applying laboratory findings to clinical trials, these limitations should be considered [139].

## 8. Clinical trials of MSC in ischemic heart disease

Various clinical trials have been executed for ischemic heart disease (coronary heart disease) with transplanted (autologous/allogeneic) mesenchymal stem cells with neither acute nor chronic myocardial infarction. Some of the completed clinical trials are listed in ***Table 1***.

**Table 1:** List of few completed clinical trials of MSC therapy in ischemic heart disease.


STUDY	n	CELL SOURCE	CONDITION	DESIGN	DELIVERY	ID NUMBER

*Chronic ischemic heart disease:*						

TRIDENT	30	Allogeneic BM	Ischemic CM	Phase II	TESI	NCT02013674

TAC-HFT	65	Autologous BM	Ischemic MI	Phase I/II	TESI	NCT00768066

PROMETHUS	9	Autologous BM	Ischemic MI	Phase I/II	IM	NCT00587990

MESAMI – I	10	Autologous BM	Ischemic CM	Phase I/II	TESI	NCT01076920

MyStromal Cell	60	Autologous ADSC	Ischemic CM	Phase II	IM	NCT01449032

Paulo et al	10	Autologous BM	Ischemic CM	Phase I/II	IC	NCT01913886

Kyriakos et al	11	Allogeneic BM	Ischemic CM	Phase II	IM	NCT01759212

POSEIDON	31	Autologous/Allogeneic BM	Ischemic CM	Phase I/II	TESI	NCT01087996

Vrtovec et al	110	Autologous BM	Ischemic dilated CM	Phase II	IC	NCT00629018

PRECISE	27	Autologous ADSC	Ischemic CM	Phase I	IM	NCT00426868

Perin et al	60	Allogeneic BM	Ischemic CM	Phase II	TSEI	NCT00721045

*Acute Myocardial infarction:*						

SEED-MC	80	Autologous BM	Acute MI	Phase II/III	IC	NCT01392105

Lian et al	160	Allogeneic UC	ST-elevation MI	Phase II	IC	NC00114452


Abbreviations: BM-Bone Marrow; ADSC- Adipose-Derived Stem Cell; UC-Umbilical Cord; CM-Cardiomyopathy; MI-Myocardial Infarction; TESI- Transendocardial stem injection; IM- intramyocardial; IC-intracoronary; MSC-Mesenchymal stem cells.

The primary clinical trial was designed as randomised research. After three months of autologous MSC derived from bone marrow intracoronary insertion in patients with acute MI, a cardiac function such as left ventricular ejection fraction developed (Chen et al., 2004). Early studies in the control of prolonged ischemic cardiomyopathy remained limited, but since 2011, several major randomised studies have produced promising results.

Hare and his co-workers [68] published the results of the POSEIDON randomised trial. Thirty patients were enrolled with LV dysfunction due to ischemic cardiomyopathy (ICM), and a couple of autologous and allogeneic BM-MSC with a mean of 150 million cells was transferred via transendocardial injection. Both types of cells had similar effects at 13 months of follow-up, such as a reduced EED (infarct size) mean of about –33.21% (95% CI, –43.61% to –22.81%; P < .001), decreased LV end-diastolic volume, and increased EF.

In the PROMETHUS trial, a total of six people with chronic ischemic cardiomyopathy leading to CABG (cardio artery bypass grafting) were injected with autologous BM via intramyocardial injection into kinetic myocardial territories. After 18 months, patients who received MSCs had a higher LV ejection fraction (+9.4± 1.7 percent, P = 0.0002) and a lower scar mass (–47.5 ±8.1 percent, P < 0.0001) than those who received placebo control. They also found that there was coherent limitation in scar size, perfusion, and enhancement in contractility [140]. However, because of the small number of participants and the deficit-managed placebo group, the notch and accuracy of these findings were limited.

The number of transplanted cells can limit the effect of MSC transplantation. Accordingly, TRIDENT conducted a safety comparative study of two doses of allogeneic BM-MSCs in ischemic cardiomyopathy. At random, thirty patients were given a transendocardial injection of either 20 million or 100 million cells. Scar size decreased equally in both groups after 12 months of follow-up: 20 thousand by –6.4 g (interquartile range, –13.5 to –3.4 g; P = 0.001) and 100 thousand by –6.1 g (interquartile range, –8.1 to –4.6 g; P = 0.0002), but the ejection fraction increased only in the 100 thousand by 3.7 U (interquartile range, 1.1 to 6.1; P = 0.04). The importance of cell doses in regenerative capacity was highlighted in this study [141]. In another study, randomised 60 patients after ischemic myocardial infarction, transplanted better with allogeneic BM transendocardially with a cell dose of about 25/75/150 × 10^6^. Three different doses were investigated, and it was discovered that a high dosage of allogeneic MSC cells was feasible and beneficial to this population [66]. Thus, the number of MSC cells transplanted into the targeted area will be more significant for better efficacy and treatment.

In addition to BM-MSCs, adipose tissue-derived mesenchymal stem cells (AD-MSCs) were used to treat ischemic cardiac disease. In a PRECISE study, in no-option patients with ischemic cardiomyopathy, AD-MSC improved total left ventricular mass, retained ventricular activity, myocardial perfusion, and contractility [142]. An ongoing phase I and II trial (UCMSC-Heart) is likely to assess the protection and virtue of umbilical cord procured mesenchymal stem cells (UC-MSCs) administered intracoronary in patients with chronic heart ischemia (Clinical Trial No: NCT02439541).

The mesenchymal cells were pretreated with growth factors and cytokines up to transplantation, which is a slightly different strategy. In the MystromalCell trial, sixty randomised patients were injected myocardially with autologous VEGF-stimulated adipose-derived MSCs (AD-MSC). Ahead of transplantation, AD-MSC was triggered to differentiate into an endothelial lineage by pre-culturing in VEGF-A165-stimulating medium for seven days. The AD-MSC group improved their exercise ability more than the placebo group.

Likely, a combination of MSCs with cardiac stem cells shows greater efficacy in treating ischemic myocardia. An ongoing clinical trial of CONCERT-HF (NCT02501811) phase II assessed the welfare, viability, and effect of autologous BM-MSCs and c-kit^+^ cardiac stem cells via transendocardial injection in patients with ischemic cardiomyopathy. A similar study, TAC-HFT-II, was designed in which the autologous hMSC and hCSC were administered transendocardially with Biosenser Webster MyoStar NOGA in patients with ischemic heart failure.

Surprisingly, there’s no comparison clinical trial study between the sources like BM-MSCs and AT-MSC to know the effect of MSCs in the treatment of any heart disease. Likewise, there are no comparative studies involving the different routes of delivery methods for this disorder. Since MSC has been demonstrated to reduce scar size, improve regional blood flow and contraction, induce vasculogenesis, reduce fibrotic effects in diseased tissue, and improve quality of life, clinical trials have some limitations, including cell dosage, the timing of cell delivery, route of administration, cell processing, and study follow-up. However, efficacy experiments on a wider scale are also unlikely to be big randomized, double-blinded, inactive controlled setups in which a huge regiment of patients could take part, so that the available information can be used for finding an optimised treatment. ***Table 2*** summarises the current clinical trials of ischemic heart disease (acute and chronic).

**Table 2:** Summary of on-going clinical trials of MSC therapy in ischemic heart disease.


STUDY	n	CELL SOURCE	CONDITION	DESIGN	DELIVERY	ID NUMBER

*Chronic ischemic heart disease:*						

MESAMI 2	90	Autologous BM	Ischemic CM	Phase II	IM	NCT02462330

HUC-Heart	79	Autologous/Allogeneic BM	Ischemic CM-Pre CABG	Phase I/II	IM	NCT02323477

UCMSC-Heart	40	Allogeneic UC	Ischemic CM, HF	Phase I/II	IC	NCT02439541

Dai et al.	45	Allogeneic UC	Ischemic CM	Phase I/II	Collagen Scaffold	NCT02635464

TAC-HFT II	55	Autologous BM ± CSC	Ischemic CM	Phase I/II	Saline	NCT02503280

SEESUPIHD	6	Allogeneic UC	Ischemic CM	Phase I/II	IC	NCT02666391

TPAABPIHD	200	Autologous BM	Ischemic CM	Phase I/II	NYD	NCT02504437

Guoping et al.	10	Allogeneic UC	Ischemic CM	Phase I	IM	NCT01946048

WJ-ICMP Tria	160	Allogeneic WJ	Ischemic CM	Phase II	IC/IV	NCT02368587

CONCERT-HF	144	Autologous BM + c-kit^+^ CSC	Ischemic CM	Phase II	IM	NCT02501811

Kyriakos et al.	5	Allogeneic BM	Ischemic CM	Phase II/III	IM	NCT01759212

Maskon et al.	80	Autologous BM	Ischemic dilated CM	Phase II	IC	NCT01720888

STEM-VAD	30	Allogeneic BM	Ischemic CM	Phase II	IV	NCT03925324

Scorem-Cells	40	Allogeneic WJ	Ischemic CM	Phase I/II	IC	NCT04011059

Harjula et al.	60	Autologous BM	Ischemic CM+CABG	Phase II	IM	NCT0041818

TEAM-AMI	124	Autologous BM	Ischemic CM	Phase II	IC	NCT0304772

*Acute Myocardial infarction:*						

Musialek et al.	115	Allogeneic BM(Cardiocell)	Acute MI	Phase II/III	IC	NCT03418233

Lien et al.	8	Allogeneic UC	Acute MI	Phase I	IC/IV	NCT04056819

PT Prodia	15	Allogeneic UC	Acute MI	Phase I/II	IC/IV	NCT04340609

ESTIMATION	50	Autologous BM	Acute MI	Phase III	IM	NCT01394432

CIRCULATE	115	Allogeneic WJ	Acute MI	Phase II/III	IC	NCT03404063

RELIEF	135	Autologous BM	Acute MI	Phase III	IC	NCT01652209

AMICI	105	Allogeneic BM	Acute MI	Phase II	IC	NCT01781390

PUMP1	60	Allogeneic BM (Provacel)	Acute MI	Phase I	IV	NCT00114452

Prochymal	220	Allogeneic BM	Acute MI	Phase II	IV	NCT00877903


Abbreviations: BM-Bone marrow; WJ-Wharton Jelly; UC-Umbilical Cord; CM-Cardiomyopathy; MI-Myocardial Infarction; TESI- Transendocardial stem injection; IM-intramyocardial; IC-intracoronary; IV-intravenous; NYD-Not Yet determined; MSC-Mesenchymal stem cells; CABG-Coronary artery bypass grafting; CSC-Cardiac stem cells.

## 9. Challenges and controversies

Mesenchymal stem cells were characterized as amongst the most highly optimistic cells for myocardial infarction and have been demonstrated to be the thriving treatment for a variety of cardiovascular diseases [143,144,145]. However, these MSC encountered many controversies and challenges that need to be addressed in future research. Some of the challenges are:

1. MSCs have the trans-differentiation potential capacity to become functional cardiac and endothelial cells [146,147,148], but their capacity to evolve into epithelial and cardiac cells *in vivo* has yet to be completely verified owing to the shortfall of unique MSC cardiac markers [149]. However, due to extracellular vesicles and exosomes, the paracrine behaviour of MSCs following implantation promotes cardiac myocyte survival, proliferation, and therapeutic properties [150,151,152].
2. There have been few reports on the possibility of proarrhythmia and cell differentiation into undesirable cell types. Eventhough Price et al reported that the possibilities of finding pro-arrhythmic electric remodelling associated with MSC treatment after infarction [10]. These multipotent stem cells transplanted into heart tissue can differentiate into noncardiac cells, which is undesirable [153]. Also, animal experimentation revealed that marrow-derived MSC administration resulted in calcification and ossification of heart tissue, besides injury to the abdominal aorta [154,155].
3. Despite numerous clinical trials involving MSCs in the treatment of cardiovascular disease, some scientists continue to question the nature of MSCs, claiming that mesenchymal stem cells cannot be distinguished based on morphology, cell surface markers, or immunological properties [156,157,158]. This demonstrates the critical nature of properly defining MSC with accurate nomenclature, as no stem cell character would be predictable in fibroblasts.
4. Despite these obstacles, the level of LVEF improvement seen with cell-based therapies is comparable to that seen with other pharmacological treatments [159]. Cellular therapies can promote cardiac repair and regenerate lost myocardial precursor cells, but they do not appear to improve myocardial contractility, according to one specific criticism. It is generally gauged as the left ventricular ejection fraction (LVEF) in patients with ischemic heart disorder. They compared that LVEF showed 2%–4% [160] and were not opposed to the distinctive effect of most commonly used pharmacological treatments. For example, beta-blockers +2.9% [161], angiotensin receptor blockade +1.3% [162], and aldosterone inhibition +2.0% [163]. So it is anticipated that improvements in stem cell therapy, such as enormous cell sources, standard delivery routes, and proper preparation protocols, will contribute to the advancement of cellular therapy in the therapeutics of cardiovascular disease.

## 10. Hurdles of MSC therapy

There are many issues that need to be addressed to enhance MSC therapy in ischemic heart disease. Clinical benefits of MSC therapy have been demonstrated in several clinical trials. However, there is substantial uncertainty because efficacy criteria for different research are not coordinated. These factors could explain why stem cell therapy has yet to be implemented in a clinical setting. Otherwise, inadequate long-term survival and integration of transplanted cells with ischemic cardiac tissue is a huge concern in stem cell-based regenerative medicine [164]. Another big hurdle is the possibility of cell-to-cell contacts between injected cells and ischemic cardiomyocytes, which could result in decreased engraftment effectiveness. Indeed, transplantation of pluripotent stem cells may result in an increase in intracellular reactive oxygen species in infarcted cardiomyocytes, which is detrimental to engraftment survival, ultimately causing cell death via paracrine or cell-autonomous mechanisms. Nonetheless, each individual reacts differently, and the outcome of any surgery is based upon the body’s ability to recuperate. Given that MSCs have a low survival rate in the cardiac environment and that a significant proportion of transplanted cells may perish shortly after transplantation, further advancements to improve MSC survival are required [165]. Shani et al. observed that the infarcted myocardium’s environment induced a proinflammatory phenotype in MSCs and inhibited their survival and paracrine impact via TLR4 [166]. Meanwhile, further papers have been released to help improve the complicated microenvironment associated with heart disease. To maximise the effects of transplanted MSCs, myocardial transfection of HIF-1 with an adenoviral vector or injection of a p38MAPK inhibitor (SB203580) was utilised. The effect of the heart disease environment on transplanted MSCs and strategies for improving the microenvironment at the transplantation site are worth further investigation in the future [67].

## 11. Future perspectives of MSC in ischemic heart disease

To promote the effective cell-based, that is, mesenchymal stem cell treatment for IHD needs optimization of the product, the use of unlimited cell source both autologous and allogeneic, delivery methods, and recipient selection. Many sophisticated methods have been introduced to increase the MSCs efficacy. They involve i) genetic manipulation (e.g., increasing engraftment potential [167], which may be effective but also harmful and non-physiological, ii) pre-conditioning *in vitro* (e.g., with hypoxia or with pharmaceutical agents) to induce their differentiation [168,169], iii) MSCs pretreatment with growth factors or cytokines (e.g., VEGF, basic FGF, and IGF-1) [170] to increase the paracrine properties, and iv) application of MSCs as microcapsules [171] and in scaffolds [172,173]. MSC functional reforms, in collaboration with the host cardiac muscle, improve the ability of cellular therapy against MI [174]. Thus the future studies are expected to reveal the latent of mesenchymal stem cells from adult tissues in the ischemic myocardial treatment.

## 12. Conclusion

Over a decade, mesenchymal cells have had a wide interest in treating cardiovascular disease owing to their prospective and differentiation. As discussed above, MSCs have a range of specific characteristics that make them more promising therapeutic agents in cell-based therapy. Rather than acting as typical stem cells which can differentiate into effector cells, they turned as governing cells that secrete mediating factors, stimulate growth conditions, or recruit those cells to perform regeneration actions in the damaged tissue. Allogeneic MSCs are particularly interested because of their ‘off-the-shelf’ therapeutic agents but are also free from fundamental limitations to autologous cells. Conclusively, many preclinical trials have shown promising results, Before MSC treatment can become a cure for a global health issue, IHD, large-scale, well-designed randomised clinical trials are required. Stem cell biology, regardless of its therapeutic potential, can hold great promise.
